# Socioeconomic and demographic determinants of children ever born among ever-married women in Somalia: a multilevel negative binomial regression analysis

**DOI:** 10.3389/frph.2026.1907831

**Published:** 2026-09-29

**Authors:** Abdirahman Osman Alin

**Affiliations:** Faculty of Health Science, Somali National University, Mogadishu, Somalia

**Keywords:** fertility, children ever born, Multilevel Negative Binomial, socioeconomic determinants, demographic factors, Somalia, reproductive health

## Abstract

Somalia has one of the highest fertility rates in the world, occurring alongside widespread socioeconomic disadvantage, low female educational attainment, and limited access to reproductive health services and information.

**Objective:**

This study examines the socioeconomic, demographic, and information-access determinants of fertility among ever-married Somali women and identifies the count-regression model that best represents total children ever born.

**Methods:**

Data were drawn from the 2020 Somali Health and Demographic Survey (SDHS), restricted to 10,772 ever-married women aged 15–49 years with complete information on all study variables. Children ever born were modeled using Poisson, Negative Binomial (NB) and Multilevel Negative Binomial (MENB) regression. Model selection was based on log-likelihood, AIC, and BIC, with comparisons restricted to models with comparable likelihood frameworks.

**Results:**

The distribution of children ever born was right-skewed, with a mean of 4.11 children and evidence of over-dispersion. The MENB model provided the best fit, with the lowest AIC (45,392.37) and BIC (45,691.04), indicating the importance of accounting for both over-dispersion and clustering. Age was the strongest predictor of fertility; women aged 45–49 years had approximately 7.5 times the expected number of children compared with those aged 15–19 years (IRR = 7.52, 95% CI: 6.94–8.14, *p* < .001). Higher education (IRR = 0.70) and higher wealth were associated with lower fertility. Significant regional differences were observed, while nomadic women had lower fertility than urban women (IRR = 0.84, *p* < .001). Divorced and widowed women also had lower fertility than currently married women. Employment, radio, and television exposure were not significant.

**Conclusion:**

Fertility in Somalia remains high but is strongly shaped by age, education, wealth, marital status and region. Expanding female education, reducing poverty, and improving access to reproductive health services and information are essential to support improve maternal and child health outcomes across Somali populations

## Introduction

Fertility outcomes are shaped by a complex interplay of socioeconomic, demographic, and regional factors across the globe. Globally, total fertility averages 2.5 children per woman, while Africa maintains a high rate of 4.7 children per woman, contributing to rapid population growth and demographic shifts, with Sub-Saharan Africa projected to account for 87% of global population growth (1, 2). Household composition, including the number of children ever born, is a critical determinant of population structure and growth (2, 3). Evidence shows that fertility is strongly influenced by female education, employment, household wealth, urbanization, and regional context, with these factors interacting to produce complex, context-specific outcomes (4–9)

Regional and urban-rural variations remain consistent predictors of fertility. Rural women in Ethiopia, for instance, have 1.37 times more children than urban women (2), a pattern echoed in Botswana, Bangladesh, and other developing contexts due to differential access to education and contraception [Kiser and Hossain (10), as cited in Cherie et al. (2)]. Multilevel analyses indicate that region and community-level factors account for significant portions of fertility variation (11, 12). In Sub-Saharan Africa, key determinants of fertility among married women include younger age, lower education for both partners, non-employment, low household wealth, non-use of contraceptives, higher ideal number of children, female-headed households, decision-making autonomy, rural residence, and regional context (13).

Educational attainment is consistently associated with lower fertility. Women with higher education have fewer children, and female education delays marriage and childbearing by increasing opportunity costs (14–16). In Ethiopia, Oman, Iran, and Kenya, higher female and paternal education reduces the number of children per household (2, 11). Socioeconomic status also strongly shapes reproductive decisions, with higher household wealth and urban residence typically linked to lower fertility [Mathenge et al. (14), as cited Rajagukguk (6)]. though effects vary across regions due to cultural norms and social support (11). Employment status, fertility intentions, and media exposure further modulated fertility (17–21). Working women generally have fewer children due to time constraints and competing responsibilities (22). Fertility is significantly influenced by ideal family size, age at first union, child mortality experiences, unmet contraceptive needs, and employment status (5, 6). Socio-cultural norms, including religion, forced pregnancy, and age at first intercourse, also shape fertility desires in Ethiopia and broader Sub-Saharan Africa (23, 24).

Environmental and macroeconomic factors additionally affect fertility trends. In the Pacific Islands and Bangladesh, higher contraceptive use, female labor force participation, urbanization, and financial factors influenced fertility decline, whereas climate shocks, child mortality, and income exerted mixed or opposing effects (7, 8). Ethnicity, marital stability, early childhood education, and housing conditions have also been shown to shape fertility outcomes (5, 9).

These patterns are not unique to the Somali context. Similar analytical approaches, including multilevel and count regression frameworks, have been widely applied in other settings such as Ethiopia and Oman to capture regional and community-level fertility variation (11, 12). More broadly, the use of count-data models to analyze fertility outcomes is well established in the global demographic literature, particularly in contexts characterized by over-dispersion and heterogeneous reproductive behavior (25).

Methodologically, fertility is commonly analyzed using count regression models because children ever born is a discrete, non-negative outcome for which ordinary least-squares regression violates distributional assumptions and can generate biased standard errors and implausible negative predictions (25). The Poisson regression model provides the conventional starting point for count data analysis because it models the probability distribution of non-negative integer outcomes. However, it assumes equality between the conditional mean and variance (equidispersion), an assumption frequently violated in demographic research where fertility counts often exhibit over-dispersion due to unobserved heterogeneity among women (25, 26). Although Poisson and Negative Binomial models account for over-dispersion, they do not explicitly evaluate whether excess zeros arise from a separate underlying process. while Multilevel Poisson models address hierarchical sampling designs, as in Oman (11). Accordingly, this study compares Poisson, Negative Binomial and Multilevel Negative Binomial models using likelihood-based criteria, including AIC and BIC, to identify the most appropriate specification for children ever born.

Media exposure and internet use are included as explanatory variables because access to information can influence reproductive knowledge, fertility preferences, and childbearing decisions. Previous research indicates that media environments shape attitudes toward family size and reproductive behavior, while digital access may influence fertility choices by expanding access to information and social opportunities (21, 27). Empirical evidence further demonstrates that digital exposure is a significant predictor of fertility preferences among reproductive-age women, highlighting the potential role of information access in shaping reproductive outcomes (28).

In the Somali community context, fertility patterns are shaped by similar determinants. Evidence from Somaliland, Somalia, and the Somali region of Ethiopia indicates that high fertility persists due to socioeconomic and demographic factors such as age, education, residence, wealth, employment, contraceptive use, and health practices, alongside cultural and medical influences including female genital mutilation (29–31). The most directly comparable national-level study is Ahmed et al. (32). That study analyzed a weighted sample of 27,352 women aged 15–49 years and, having detected over-dispersion, estimated a single survey-adjusted Negative Binomial regression to obtain adjusted incidence rate ratios for demographic, socioeconomic, reproductive, and geographic covariates; it reported that higher education, later age at first birth, literacy, nomadic residence, and modern contraceptive use were associated with fewer children ever born, while urban residence and middle wealth status were associated with more (32). in terms of methodology (32,) estimated a single model (Negative Binomial) chosen because of over-dispersion alone, without formally testing whether the zero counts themselves reflect a distinct structural process; In contrast, this study adopts a comparative count-model framework, estimating: Poisson, Negative Binomial and Multilevel Negative Binomial. Model selection is based on log-likelihood, AIC, and BIC.

Third, the variable sets differ: (32) foreground reproductive and behavioral variables such as age at first birth and contraceptive-use intentions, whereas the present study foregrounds media exposure, internet use, and employment status alongside the standard socioeconomic and demographic covariates, allowing an explicit test of whether information-access variables retain explanatory power once demographic and wealth are accounted for. Fourth, in terms of contribution, this study extends the descriptive, single-model estimation approach of (32) toward a methodological framework for model selection in high-fertility, low-information settings, offering guidance that is transferable to other fragile or data-limited contexts beyond Somalia. This study utilizes the Somali Health and Demographic Survey to assess the influence of age, education, region, residence, wealth, employment, marital status, internet and media exposure on the number of children ever born, It applies a comparative count-regression approach, culminating in a multilevel negative binomial model, which simultaneously accounts for: over-dispersion, clustering (enumeration areas), situating Somali fertility trends within broader Sub-Saharan African and global contexts.

Despite the growing literature on fertility determinants in Somalia and related Somali populations, several important gaps remain. First, empirical evidence focusing specifically on cumulative fertility experience measured as children ever born, among Somali women remains limited and methodologically narrow. Previous Somali studies have examined fertility intentions using multinomial logistic regression (29), fertility preference using machine-learning classification (28), and children ever born using a single Negative Binomial regression model (32); Although these studies provide important evidence on fertility-related outcomes, no previous Somali study has systematically compared alternative count-data models to determine the most appropriate statistical specification for modeling children ever born. Second, previous research has frequently examined fertility determinants through separate socioeconomic, demographic, behavioral, or information-access domains rather than integrating these factors within a unified analytical framework. Determinants such as age, education, household wealth, residence, employment, marital status, media exposure, and internet use may interact and confound one another. Examining these factors simultaneously is therefore necessary to understand their independent associations with fertility after accounting for competing influences. Third, an important methodological gap concerns the treatment of the statistical characteristics of fertility count data.

### Problem statement

Fertility in Somalia remains among the highest globally, yet existing analytical approaches have not fully addressed the complex statistical and contextual characteristics of fertility data. Previous studies have primarily relied on single-model approaches, limiting understanding of whether alternative count-regression specifications provide better representation of children ever born. In addition, limited attention has been given to community-level clustering, despite the fact that women surveyed in demographic studies are nested within enumeration areas with potentially shared fertility norms and socioeconomic environments.

Building on these gaps, this study addresses the following research questions:

1. Which count-regression model provides the best statistical representation of total children ever born among ever-married Somali women aged 15–49 years?
2. Which demographic, socioeconomic, geographic, and information-access factors including age, region, residence, education, wealth, marital status, employment, media exposure, and internet use are significantly associated with the number of children ever born?
3. How do the findings of this study compare with previous national evidence on children ever born in Somalia, and what do differences in sample construction and modeling strategies imply for understanding fertility determinants in Somalia?

### Novelty and contribution

This study contributes to Somali fertility research in three ways. First, it empirically compares Poisson, Negative Binomial and Multilevel Negative Binomial models using the same dataset to identify the most appropriate specification for children ever born. Second, it improves methodological understanding by accounting for over-dispersion and enumeration-area clustering through multilevel modeling and model selection using AIC and BIC. Third, it extends previous evidence, by examining the robustness of fertility determinants under different modeling and analytical approaches, providing relevant evidence for fertility research in data-limited settings.

## Methodology

This study investigates the socioeconomic and demographic determinants of fertility using count regression models to analyze women's reproductive outcomes. The study uses secondary data obtained from Somali Demographic and Health Surveys (SDHS).

### Study population and variables

The analysis was restricted to ever-married women aged 15–49 years (currently married, divorced, or widowed). Of the 16,486 women aged 15–49 interviewed, 11,876 were ever-married and eligible. A complete-case restriction on the study variables further excluded 1,104 women (9.3%), yielding the final analytical sample of 10,772 (Figure 1). All findings and conclusions in this manuscript pertain to ever-married women and should not be generalized to never-married women.

**Figure 1:**
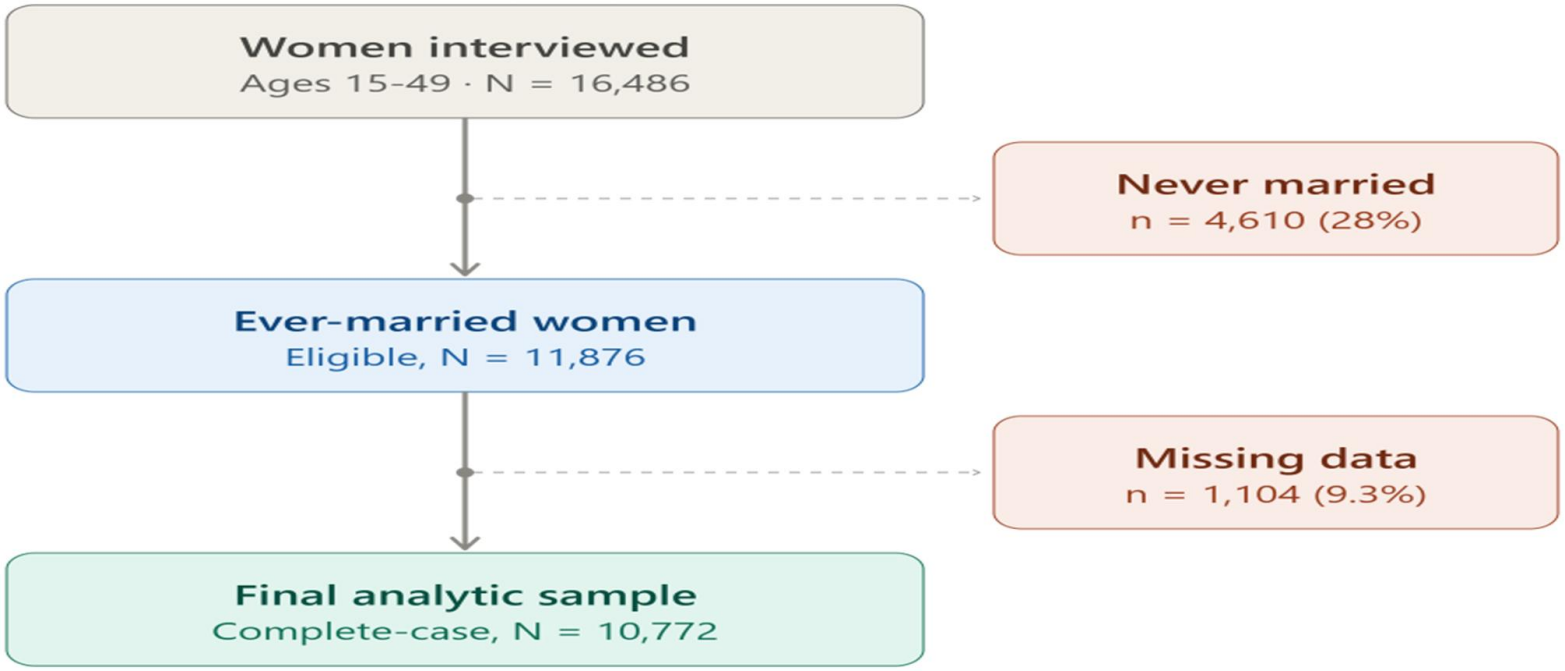
Flow diagram of study population derivation, Somali demographic and health survey.

#### Survey design and weighting

The Somali Demographic and Health Survey (SDHS 2020) use a complex, multistage sampling design incorporating stratification, clustering, and unequal selection probabilities. The analysis did not apply survey weights; however, clustering at the enumeration-area level (V001) was explicitly accounted for in the multilevel modeling framework.

#### Outcome variable

The dependent variable is total children ever born, denoted by $\;Y_{i}$, measured using SDHS variable V201.

#### Explanatory variables

The explanatory variables include Variables included, Age group (V013), Region (V101), Residence, (V102), Education (V106), Wealth quintile (V190), Marital status (V501), Employment (V731), Media exposure (V158, V159), Internet use (V171). Observations with missing values in any of these variables were excluded.

#### Descriptive analysis

Descriptive statistics were computed to summarize the characteristics of the study population. Frequencies and percentages were reported for categorical variables, while the distribution of total children ever born was examined using summary statistics and graphical analysis. histograms and kernel density plots to evaluate skewness and dispersion (33). Because fertility is measured as a non-negative integer count, the exploratory analysis was used to assess whether count regression models were more appropriate than ordinary linear regression (25).

#### Count regression modeling framework

Let $\;Y_{i}$ denote the total number of children ever born to woman *i*, and let $X_{i}$ represent a vector of socioeconomic and demographic covariates. The conditional mean of the outcome variable is defined a

$$\mu_{i}=E(Y_{i}|X_{i})$$ (1)

Count regression models relate the expected fertility count to explanatory variables using log-link functions;

$$log(\mu_{i})=X_{i}^{⊤}\beta ,$$ (2)

Where *β* is a vector of parameters to be estimated, this specification ensures non-negative predicted values and allows covariates to exert multiplicative effects on fertility outcomes (25).

#### Poisson regression model

$$Y_{i}∼Poisson(\mu_{i})$$

$$P(Y_{i}\;=\;y_{i})=\;\frac{e^{-\mu_{i}}\mu_{i}^{y_{i}}}{y_{i}!},y_{i}=0,\;1,\;2,\;3\ldots$$ (3)

With the defining (equidispersion) assumption:

$$E(Y_{i}|X_{i})=V(Y_{i}|X_{i})=\mu_{i}$$ (4)

However, this assumption is often violated in fertility data due to over-dispersion an assumption that is often restrictive in demographic applications, since fertility counts frequently exhibit over-dispersion, where the variance exceeds the mean (25).

#### Negative binomial model

To address over-dispersion, the negative binomial model introduces a dispersion parameter *α*. The outcome is assumed to follow a negative binomial distribution:

$$P(y_{i}|\mu i,\alpha )=\frac{\Gamma (y_{i}+\alpha^{-1})}{\Gamma (y_{i}+1)\Gamma (\alpha^{-1})}\;{\left(\frac{\alpha^{-1}}{\alpha^{-1}+\mu i}\right)}^{\alpha^{-1}}{\left(\frac{\mu i}{\alpha^{-1}+\mu_{i}}\right)}^{y_{i}}$$ (5)

With conditional mean and variance:

$$V(Y_{i}|X_{i})=\mu_{i}+\alpha \mu_{i}^{2}$$ (6)

Where *α* > 0 indicates over-dispersion (34).

#### Multilevel negative binomial model

To account for clustering of women within enumeration areas, a multilevel negative binomial model with random intercepts was estimated:

$$log(\mu_{ij})=X_{ij}^{⊤}\beta +u_{j}$$ (7)

Where: i = individual and j = cluster (enumeration area, V001)

#### Model selection and estimation

Model performance was compared using the log-likelihood, Akaike Information Criterion (AIC), and Bayesian Information Criterion (BIC) (35, 36). The final model was selected based on statistical fit and substantive interpretability. All statistical analyses were conducted using Stata version 17 (37).

## Results

Table 1 presents the descriptive characteristics of the study population on a sample of 10,772 women. The distribution of total children ever born (V201) indicates varying fertility levels, with the largest proportions of women having 3 children (13.44%), followed by 2 children (12.63%) and 4 children (12.31%). About 10.26% of women reported having no children, while very high fertility (10 or more children) is relatively rare. In terms of age distribution (V013), the majority of respondents fall within the 25–29 age group (23.67%), followed by 20–24 (19.11%) and 30–34 (18.14%), indicating a predominantly young reproductive population. Regionally (V101), respondents are fairly distributed across regions, with the highest representation from Banadir (13.31%), while regions such as Bay (2.14%) have smaller shares.

**Table 1:** Descriptive statistics of study variables (*N* = 10,772).

Variable	Category	Frequency	Percent (%)
Total Children Ever Born (V201)	0	1,105	10.26
	1–2	2,499	23.19
	3–4	2,774	25.75
	5–6	2,184	20.28
	7–8	1,322	12.27
	9–10	624	5.80
	11+	264	2.45
	Total	10,772	100.00
Age Group (V013)	15–19	981	9.11
	20–24	2,059	19.11
	25–29	2,550	23.67
	30–34	1,954	18.14
	35–39	1,699	15.77
	40–44	987	9.16
	45–49	542	5.03
	Total	10,772	100.00
Region (V101)	Awdal	513	4.76
	Woqooyi Galbeed	833	7.73
	Togdheer	890	8.26
	Sool	831	7.71
	Sanaag	903	8.38
	Bari	510	4.73
	Nugaal	515	4.78
	Mudug	637	5.91
	Galgaduud	580	5.38
	Hiraan	621	5.76
	Middle Shabelle	506	4.70
	Banadir	1,434	13.31
	Bay	231	2.14
	Bakool	499	4.63
	Gedo	591	5.49
	Lower Juba	678	6.29
	Total	10,772	100.00
Residence (V102)	Urban	4,607	42.77
	Rural	2,852	26.48
	Nomadic	3,313	30.76
	Total	10,772	100.00
Education (V106)	No Education	9,020	83.74
	Primary	1,235	11.46
	Secondary	389	3.61
	Higher	128	1.19
	Total	10,772	100.00
Wealth Quintile (V190)	Lowest	2,368	21.98
	Second	2,316	21.50
	Middle	2,002	18.59
	Fourth	2,066	19.18
	Highest	2,020	18.75
	Total	10,772	100.00
Marital Status (V501)	Married	9,423	87.48
	Divorced	897	8.33
	Widowed	452	4.20
	Total	10,772	100.00
Employment (V731)	Yes	159	1.48
	No	10,613	98.52
	Total	10,772	100.00
Radio Exposure (V158)	At least weekly	669	6.21
	Less than weekly	287	2.66
	Not at all	9,816	91.13
	Total	10,772	100.00
Television Exposure (V159)	At least weekly	961	8.92
	Less than weekly	294	2.73
	Not at all	9,517	88.35
	Total	10,772	100.00
Internet Use (V171)	Yes	1,202	11.16
	No	9,570	88.84
	Total	10,772	100.00

Media exposure is generally low. Most women reported not listening to the radio (91.13%) or watching television (88.35%) at all. Similarly, internet use (V171) is limited, with only 11.16% reporting usage. Educational attainment (V106) is predominantly low, with 83.74% of women having no formal education, while only 1.19% attained higher education. The wealth distribution (V190) is relatively balanced across quintiles, though a slightly higher proportion falls in the lowest category (21.98%).

Regarding place of residence (V102), 42.77% of respondents live in urban areas, 26.48% in rural areas, and 30.76% are nomadic, reflecting Somalia's diverse settlement patterns. Most women are currently married (87.48%), with smaller proportions divorced (8.33%) or widowed (4.20%). Employment levels (V731) are extremely low, with only 1.48% of women reporting being employed.

Overall, the descriptive statistics highlight a population characterized by high fertility, low educational attainment, limited media exposure, and low female employment, which are key factors influencing reproductive outcomes in Somalia.

The distribution of children ever born in Figure 2 was markedly right-skewed, with the majority of women reporting between 1 and 6 children and a long thin tail extending to 17–18. This skew, together with a variance substantially exceeding the mean, indicated over dispersion in the outcome and motivated comparison of Poisson, negative binomial, and multilevel negative binomial specifications (Table 4), where the negative binomial models showed substantially improved fit over Poisson.

**Figure 2:**
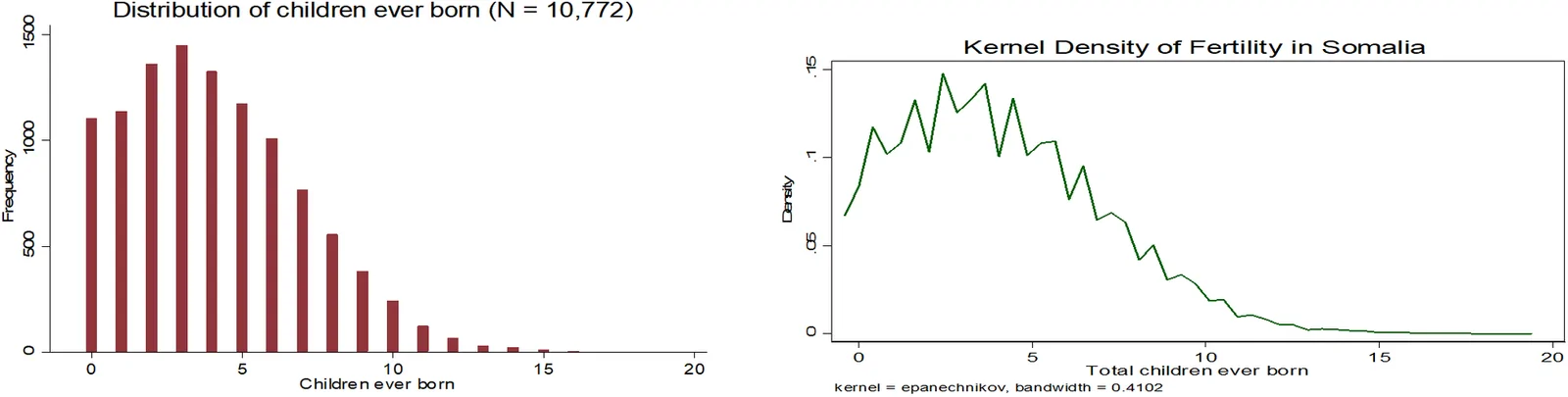
Distribution of children ever born (histogram, *N* = 10,772).

Table 2 shows that women reported a mean of 4.11 children ever born (SD = 2.92). Fertility increased with age, from 0.83 among women aged 15–19 to 6.43 among those aged 45–49. Mean fertility declined with higher education, from 4.26 among women with no education to 2.00 among those with higher education, and was slightly lower among wealthier women. Regional and marital-status differences were also evident. Internet users had substantially fewer children (2.88) than non-users (4.27), while radio, television, residence, and employment showed relatively small differences. Overall, the descriptive findings suggest variation in fertility by age, education, wealth, marital status, region, and internet use. However, these are unadjusted associations; the subsequent multilevel negative binomial regression assesses whether these relationships remain after adjustment for other factors.

**Table 2:** Distribution of total children ever born by selected characteristics among ever-married women aged 15–49 years, Somalia (*N* = 10,772).

Variable	Category	*N*	Mean	SD	Median	IQR (p25–p75)
Age group	15–19	981	0.83	0.94	1	0–1
	20–24	2,059	2.14	1.47	2	1–3
	25–29	2,550	3.66	2.04	4	2–5
	30–34	1,954	5.00	2.54	5	3–7
	35–39	1,699	6.11	2.78	6	4–8
	40–44	987	6.19	3.05	6	4–8
	45–49	542	6.43	3.44	6	4–9
Region	Awdal	513	3.69	2.80	3	1–6
	Woqooyi Galbeed	833	3.92	2.89	4	2–6
	Togdheer	890	4.33	3.21	4	2–6
	Sool	831	4.32	2.94	4	2–6
	Sanaag	903	4.17	3.06	4	2–6
	Bari	510	3.99	2.91	4	2–6
	Nugaal	515	4.05	2.95	4	2–6
	Mudug	637	3.83	2.93	3	2–6
	Galgaduud	580	4.65	3.07	4	2–7
	Hiraan	621	4.21	2.71	4	2–6
	Middle Shabelle	506	3.93	2.84	4	2–6
	Banadir	1,434	4.11	2.90	4	2–6
	Bay	231	4.27	2.74	4	2–6
	Bakool	499	4.01	2.65	4	2–6
	Gedo	591	4.15	2.93	4	2–6
	Lower Juba	678	4.01	2.68	4	2–6
Residence	Urban	4,607	4.12	2.94	4	2–6
	Rural	2,852	4.29	2.97	4	2–6
	Nomadic	3,313	3.95	2.84	4	2–6
Education	No education	9,020	4.26	2.92	4	2–6
	Primary	1,235	3.61	2.82	3	1–5
	Secondary	389	3.00	2.71	2	1–5
	Higher	128	2.00	1.95	1	1–3
Wealth quintile	Lowest	2,368	4.21	2.90	4	2–6
	Second	2,316	4.21	2.87	4	2–6
	Middle	2,002	4.28	2.93	4	2–6
	Fourth	2,066	4.06	2.95	4	2–6
	Highest	2,020	3.76	2.93	3	1–6
Marital status	Married	9,423	4.21	2.92	4	2–6
	Divorced	897	2.81	2.50	2	1–4
	Widowed	452	4.58	3.08	4	2–7
Employment status	Worked in last 12 months	159	4.39	3.14	4	2–6
	Did not work	10,613	4.11	2.92	4	2–6
Radio exposure	At least once a week	669	4.14	2.95	4	2–6
	Less than once a week	287	3.72	3.05	3	1–5
	Not at all	9,816	4.12	2.91	4	2–6
Television exposure	At least once a week	961	3.72	2.94	3	1–5
	Less than once a week	294	3.70	3.11	3	1–6
	Not at all	9,517	4.16	2.91	4	2–6
Internet use	Yes	1,202	2.88	2.58	2	1–4
	No	9,570	4.27	2.92	4	2–6
Total	—	10,772	4.11	2.92	4	2–6

Model performance was evaluated using AIC and BIC, where lower values indicate better fit (see Table 3). Among the comparable models (Poisson, NB, and MENB), the multilevel negative binomial (MENB) model showed the best fit, with the lowest AIC (45,392.37) and BIC (45,691.04), indicating the importance of accounting for both over dispersion and clustering.

**Table 3:** Model comparison using information criteria.

Model	Log Likelihood	AIC	BIC	Interpretation
Poisson	−22756.74	45591.48	45875.58	Poor fit; ignores overdispersion
Negative Binomial	−22666.88	45413.76	45705.15	Improved; accounts for overdispersion
Multilevel NB (MENB)	−22655.18	45392.37	45691.04	Best fit; accounts for clustering and overdispersion

The results from both the Negative Binomial (NB) and Multilevel Negative Binomial (MENB) models are highly consistent, indicating robust findings (Table 4). Age is the strongest predictor of total children ever born; with women aged 45–49 having nearly 8 times higher fertility compared to those aged 15–19. Regional differences are significant, with most regions showing higher fertility relative to Awdal.

**Table 4:** Comparison of incidence rate ratios (IRR) from NB and multilevel NB (MENB) model of children ever born.

Variable	Category	NB IRR (95% CI)	MENB IRR (95% CI)
Age group (Ref: 15–19)	20–24	2.597*** (2.406–2.804)	2.603*** (2.411–2.810)
	25–29	4.409*** (4.097–4.746)	4.422*** (4.109–4.760)
	30–34	6.009*** (5.582–6.468)	6.029*** (5.600–6.490)
	35–39	7.316*** (6.796–7.876)	7.338*** (6.817–7.900)
	40–44	7.599*** (7.042–8.200)	7.627*** (7.069–8.230)
	45–49	7.919*** (7.307–8.581)	7.962*** (7.347–8.628)
Region (Ref: Awdal)	Woqooyi Galbeed	1.117*** (1.049–1.189)	1.119*** (1.047–1.196)
	Togdheer	1.251*** (1.177–1.329)	1.251*** (1.173–1.335)
	Sool	1.309*** (1.231–1.392)	1.313*** (1.230–1.403)
	Sanaag	1.280*** (1.204–1.361)	1.274*** (1.193–1.361)
	Bari	1.215*** (1.133–1.303)	1.207*** (1.120–1.300)
	Nugaal	1.296*** (1.209–1.390)	1.293*** (1.200–1.393)
	Mudug	1.203*** (1.126–1.286)	1.226*** (1.141–1.317)
	Galgaduud	1.482*** (1.387–1.583)	1.468*** (1.367–1.578)
	Hiraan	1.351*** (1.265–1.444)	1.345*** (1.253–1.444)
	Middle Shabelle	1.381*** (1.286–1.483)	1.370*** (1.272–1.476)
	Banadir	1.381*** (1.300–1.466)	1.372*** (1.286–1.463)
	Bay	1.236*** (1.133–1.349)	1.232*** (1.119–1.356)
	Bakool	1.210*** (1.128–1.297)	1.211*** (1.124–1.305)
	Gedo	1.256*** (1.175–1.343)	1.257*** (1.170–1.350)
	Lower Juba	1.220*** (1.143–1.303)	1.216*** (1.133–1.306)
Education (Ref: No education)	Primary	1.073*** (1.034–1.113)	1.074*** (1.035–1.114)
	Secondary	1.001 (0.934–1.072)	0.999 (0.933–1.071)
	Higher	0.689*** (0.602–0.789)	0.694*** (0.607–0.795)
Wealth quintile (Ref: Lowest)	Second	0.971 (0.940–1.004)	0.977 (0.944–1.011)
	Middle	0.925*** (0.887–0.964)	0.923*** (0.884–0.963)
	Fourth	0.878*** (0.841–0.917)	0.878*** (0.839–0.919)
	Highest	0.839*** (0.801–0.880)	0.842*** (0.801–0.885)
Residence (Ref: Urban)	Rural	0.969* (0.940–0.999)	0.988 (0.947–1.030)
	Nomadic	0.853*** (0.820–0.887)	0.849*** (0.812–0.887)
Marital status (Ref: Married)	Divorced	0.705*** (0.674–0.736)	0.706*** (0.676,0.738)
	Widowed	0.879*** (0.836–0.925)	0.879*** (0.836,0.924)
Employment (Ref: Yes)	No	1.042 (0.958–1.133)	1.044 (0.961–1.135)
Radio exposure (Ref: Weekly)	Less than weekly	0.990 (0.914–1.073)	0.990 (0.914–1.073)
	Not at all	0.987 (0.943–1.033)	0.993 (0.948–1.039)
TV exposure (Ref: Weekly)	Less than weekly	1.001 (0.927–1.082)	0.997 (0.923–1.077)
	Not at all	0.983 (0.939–1.029)	0.982 (0.938–1.029)
Internet use (Ref: Yes)	No	1.147*** (1.096–1.201)	1.146*** (1.095–1.200)
Model fit	Observations	10,772	10,772
	Log likelihood	−22,666.88	−22,655.18
	AIC	45,413.76	45,392.37
	BIC	45,705.15	45,691.04

*p < 0.05; **p < 0.01; ***p < 0.001.

Education is negatively associated with fertility; women with higher education have significantly fewer children. Similarly, higher wealth status is linked to lower fertility, suggesting a socioeconomic gradient. Nomadic populations exhibit significantly lower fertility than urban residents, while rural differences are weak.

Marital status matters, divorced and widowed women tend to have fewer children compared to married women. Access to health services is associated with higher reported fertility, while contraceptive use and media exposure show no significant effects.

The multilevel negative binomial regression in Table 5 showed that age was the strongest predictor of total children ever born. Compared with women aged 15–19 years, the expected number of children was progressively higher with age, reaching an IRR of 7.52 (95% CI: 6.94–8.14) among women aged 45–49 years (*p* < 0.001).

**Table 5:** Multilevel negative binomial regression analysis of factors associated with total children ever born among ever-married women aged 15–49 years, Somalia (*N* = 10,772).

Variables	MENB: IRR (95% CI)
Age group	
15–19 (Ref.)	1.000
20–24	2.601*** (2.415–2.806)
25–29	4.412*** (4.101–4.745)
30–34	5.957*** (5.539–6.411)
35–39	7.223*** (6.714–7.771)
40–44	7.465*** (6.923–8.050)
45–49	7.515*** (6.937–8.141)
Region	
Awdal (Ref.)	1.000
Woqooyi Galbeed	1.119*** (1.049–1.194)
Togdheer	1.229*** (1.154–1.305)
Sool	1.288*** (1.208–1.373)
Sanaag	1.268*** (1.189–1.352)
Bari	1.180*** (1.097–1.270)
Nugaal	1.284*** (1.194–1.380)
Mudug	1.216*** (1.133–1.304)
Galgaduud	1.457*** (1.359–1.561)
Hiraan	1.337*** (1.248–1.433)
Middle Shabelle	1.357*** (1.267–1.465)
Banadir	1.366*** (1.278–1.455)
Bay	1.230*** (1.119–1.350)
Bakool	1.214*** (1.130–1.305)
Gedo	1.236*** (1.154–1.327)
Lower Juba	1.220*** (1.134–1.307)
Education	
No education (Ref.)	1.000
Primary	0.931*** (0.898–0.965)
Secondary	0.997 (0.931–1.067)
Higher	0.703*** (0.615–0.802)
Wealth index	
Lowest (Ref.)	1.000
Second	0.974 (0.946–1.007)
Middle	0.913*** (0.875–0.952)
Fourth	0.865*** (0.827–0.904)
Highest	0.843*** (0.803–0.885)
Residence	
Urban (Ref.)	1.000
Rural	0.985 (0.946–1.026)
Nomadic	0.842*** (0.806–0.879)
Marital status	
Married (Ref.)	1.000
Divorced	0.711*** (0.681–0.742)
Widowed	0.875*** (0.833–0.919)
Employment	
Yes (Ref.)	1.000
No	1.074 (0.988–1.167)
Radio exposure	
At least once a week (Ref.)	1.000
Less than once a week	0.976 (0.902–1.056)
Not at all	0.990 (0.946–1.035)
TV exposure	
At least once a week (Ref.)	1.000
Less than once a week	1.011 (0.938–1.091)
Not at all	1.004 (0.959–1.050)
Internet Use	
Yes (Ref.)	1.000
No	1.147*** (1.097–1.200)
Observations	10,700
Groups (V001)	316
Wald *χ*^2^ (38)	7,040.31
Prob > χ^2^	<0.001
Log likelihood	−22,262.681
Random-effect variance	0.00444
LR test vs. NB	χ¯^2^ (01) = 20.31, *p* < 0.001

*p < 0.05; **p < 0.01; ***p < 0.001.

Significant regional differences were observed, with fertility counts higher in all regions compared with Awdal, particularly Galgaduud (IRR = 1.46), Sool (1.29), and Nugaal (1.28). Higher education was associated with lower fertility; women with higher education had approximately 30% fewer children than those with no education (IRR = 0.70, 95% CI: 0.62–0.80). The inverse association between higher education and children ever born suggests that advanced educational opportunities may contribute to delayed childbearing and lower cumulative fertility experience.

Similarly, women in the middle, fourth, and highest wealth categories had significantly lower fertility than those in the lowest wealth category.

Nomadic women had lower fertility than urban women (IRR = 0.84), while divorced and widowed women also had lower counts than married women. Internet non-use was associated with 15% higher fertility (IRR = 1.15, 95% CI: 1.10–1.20). Employment, radio exposure, and TV exposure were not significantly associated with the number of children ever born.

The overall model was statistically significant (Wald *χ*^2^ = 7,040.31, *p* < 0.001). Importantly, the LR test comparing the mixed-effects model with the standard negative binomial model was significant (χ¯^2^ = 20.31, *p* < 0.001), supporting the inclusion of the cluster-level random effect and therefore the use of the multilevel/mixed-effects negative binomial model.

## Discussion

This study identified several important demographic, socioeconomic, geographic, and information-related factors associated with children ever born among ever-married Somali women aged 15–49 years. The strongest finding was the marked association between age and cumulative fertility. Compared with women aged 15–19 years, the expected number of children increased progressively across age groups, reaching an IRR of 7.515 among women aged 45–49 years. This finding is consistent with previous studies from Sub-Saharan Africa (2, 13). Because children ever born are a cumulative measure, the strong age effect likely reflects longer exposure to marriage and childbearing over the reproductive life course rather than a direct causal effect of age.

The inverse association between higher education and fertility was another important finding and is consistent with previous evidence from Somalia and other developing settings (2, 11, 15). Women with higher education had substantially fewer children than women with no education. This pattern supports the demographic transition perspective that education may delay marriage and childbearing and increase the opportunity costs of having larger families (4). Similarly, women in higher wealth categories generally had lower fertility, supporting evidence that socioeconomic conditions are important in shaping reproductive behavior (4, 6).

Regional variation was also a strong and consistent finding. Fertility was significantly higher in all regions compared with Awdal, with particularly large differences in Galgaduud, Hiraan, Sool, and Nugaal. This finding is consistent with multilevel evidence showing that geographic and community contexts contribute substantially to fertility differences (11, 12).

A notable finding that differed from some previous African studies was the lower fertility among nomadic women compared with urban women. Previous research often reports higher fertility among rural populations than urban populations (2, 7, 8). However, this finding is broadly consistent with the national evidence reported by (32) which also identified nomadic residence as associated with fewer children ever born. Differences in livelihood, mobility, marriage patterns, and access to services may contribute to this distinctive Somali pattern.

The findings concerning information access were mixed. Employment, radio, and television exposure were not significantly associated with children ever born, whereas internet non-use was significantly associated with higher fertility. The latter finding is consistent with emerging evidence that digital access may influence reproductive information, preferences, and behavior (21, 27, 28).

Overall, the findings indicate that age, education, wealth, and regional context are the most important factors associated with cumulative fertility, while residence and internet access provide additional evidence of context-specific fertility patterns in Somalia. The results are generally consistent with previous literature but also demonstrate important differences, particularly regarding nomadic residence and information access. The study further contributes methodologically by using a multilevel negative binomial model that accounts for both over-dispersion and clustering of women within enumeration areas, addressing an important gap in previous Somali fertility research

### Limitations

The study is limited by its cross-sectional design, lack of survey weighting, and potential selection bias from missing data. In addition, the findings apply only to ever-married women aged 15–49 years. Finally, using children ever born does not capture other fertility dimensions such as birth spacing, fertility intentions, or contraceptive behavior.

### Future research

Future studies should use longitudinal and survey-weighted data to strengthen causal interpretation and population-level estimates. Further research should examine regional and nomadic fertility differences, as well as the role of internet and digital reproductive-health information. Future studies should also include fertility intentions, birth spacing, contraceptive behavior, age at marriage, and age at first birth to provide a more complete understanding of fertility patterns.

## Conclusion and recommendations

The findings of this study are consistent with the demographic transition hypothesis, which describes the shift from high to low fertility as countries undergo socioeconomic development, with improvements in education, income, and access to healthcare contributing to changes in reproductive behavior. In the Somali context, fertility patterns are closely linked to socioeconomic and demographic conditions, particularly Age, education, wealth, and regional disparities.

From a policy perspective, this study does not advocate fertility reduction as an isolated objective, but on improving maternal and child health through enhanced education, economic conditions, and access to reproductive health services. Supporting informed and voluntary reproductive choices is essential, ensuring that fertility decisions are guided by socioeconomic readiness. Strengthening female education and improving healthcare access therefore remain central to promoting healthy fertility patterns and better maternal and child health outcomes in Somalia.

## Data Availability

Publicly available datasets were analyzed in this study. This data can be found here: https://microdata.nbs.gov.so/index.php/catalog/50.

## References

[1] Lam D Leibbrandt M Allen J. The Demography of the Labor Force in Sub-Saharan Africa: Challenges and Opportunities. Bonn: IZA – Institute of Labor Economics (2019).

[2] Cherie N Getacher L Belay A Gultie T Mekuria A Sileshi S. Modeling on number of children ever born and its determinants among married women of reproductive age in Ethiopia: a Poisson regression analysis. Heliyon. (2023) 9(3):e13948. 10.1016/j.heliyon.2023.e13948 36879961 PMC9984836

[3] Esteve A Pohl M Becca F Fang H Galeano J García-Román J. A global perspective on household size and composition, 1970–2020. Genus. (2024) 80(1):1–22. 10.1186/s41118-024-00211-6 PMC10830558 38296987

[4] Bongaarts J Mensch BS Blanc AK. Trends in the age at reproductive transitions in the developing world: the role of education. Popul Stud (NY). (2017) 71(2):139–54. 10.1080/00324728.2017.1291986 28397543

[5] Golub R Ivkov-Dzigurski A Simeunović V. Determinants of fertility intentions of the women in Bosnia and Herzegovina—an example from the Semberija region. Behav Sci (Basel). (2023) 13(5):417. 10.3390/bs13050417 37232653 PMC10215066

[6] Rajagukguk W. Socioeconomic and proximate determinants of high fertility in Timor-Leste. J Econ Bus. (2023) 6(4):230–43. 10.31014/aior.1992.06.04.549

[7] Lal S Singh R Makun K Chand N Khan M. Socio-economic and demographic determinants of fertility in six selected Pacific island countries: an empirical study. PLoS One. (2021) 16(9):1–17. 10.1371/journal.pone.0257570 PMC8457485 34550992

[8] Atiqul Haq SM Chowdhury MAB Ahmed KJ Uddin MJ. Effects of extreme climate events and child mortality on total fertility rate in Bangladesh. Heliyon. (2024) 10(15):e35087. 10.1016/j.heliyon.2024.e35087 39170491 PMC11336454

[9] Nestorová Dická J Lipták F. Regional fertility predictors based on socioeconomic determinants in Slovakia. J Popul Res. (2024) 41:1–43. 10.1007/s12546-024-09340-3

[10] Kiser H Hossain MA. Estimation of number of ever born children using zero truncated count model: evidence from Bangladesh demographic and health survey. Heal Inf Sci Syst. (2018) 7(1):3. 10.1007/s13755-018-0064-y PMC6281539 30588293

[11] Al-Balushi MS Ahmed MS Islam MM Khan MHR. Multilevel Poisson regression modeling to identify factors influencing the number of children ever born to married women in Oman. J Stat Manag Syst. (2020) 23(8):1357–73. 10.1080/09720510.2019.1709328

[12] Tessema NS Geda NR. Trends in maternal education - based inequalities in under - five mortality in Ethiopia: multilevel, decomposition and concentration index analyses. Discov Soc Sci Heal. (2024) 4:. 10.1007/s44155-024-00122-z

[13] Teshale AB Worku MG Tesema GA. Fertility decision and its associated factors in Sub-Saharan Africa: a multilevel multinomial logistic regression analysis. BMC Womens Health. (2022) 22(1):1–11. 10.1186/s12905-022-01920-w 35941648 PMC9358800

[14] Mathenge W Foster A Kuper H. Urbanization, ethnicity and cardiovascular risk in a population in transition in Nakuru, Kenya: a population-based survey. BMC Public Health. (2010) 10:15–7. 10.1186/1471-2458-10-569 20860807 PMC2956724

[15] Radovich E el-Shitany A Sholkamy H Benova L. Rising up: fertility trends in Egypt before and after the revolution. PLoS One. (2018) 13(1):1–14. 10.1371/journal.pone.0190148 PMC5773010 29346389

[16] Yaukey D. Trends and variations in fertility in the United States. By Clyde V. Kiser, Wilson H. Grabill, and Arthur A. Campbell. Cambridge: Harvard University Press, 1968. 338 pp. $6.95. Soc Forces. (1969) 47(4):495–6. 10.2307/2574562

[17] Ning C Wu J Ye Y Yang N Pei H Gao H. How media use influences the fertility intentions among Chinese women of reproductive age: a perspective of social trust. Front Public Health. (2022) 10:1–12. 10.3389/fpubh.2022.882009 PMC9127136 35619808

[18] Shah IA. The link between employment stability and fertility intention: a cross sectional study. Int J Sociol Anthropol. (2018) 10:10–20.

[19] Molino M Sacchi A Vaccargiu F Ghislieri C. The intersection of work, career, and parenthood: a qualitative exploration of fertility intentions in the Italian context. Soc Sci. (2026) 2024(Oecd 2024):1–24. 10.3390/socsci15070434

[20] Hanappi D Ryser V-A Bernardi L Le Goff J-M. Changes in employment uncertainty and the fertility intention—realization link: an analysis based on the Swiss household panel. Eur J Popul. (2017) 33:381–407. 10.1007/s10680-016-9408-y 28725099 PMC5493711

[21] Hadadian F Abbasi M Parsajam T Haseli A Rostami N. Screens and society: how media narratives influence fertility and reshape childbearing choices; a systematic review. BMC Womens Health. (2025) 25:1–15. 10.1186/s12905-025-03986-8 . 41024121 PMC12482673

[22] Matysiak A Vignoli D. Diverses conséquences de l’emploi des femmes sur la fécondité: quelques informations à partir des cas de l’Italie et de la Pologne. Eur J Popul. (2013) 29(3):273–302. 10.1007/s10680-013-9287-4 23956480 PMC3744382

[23] Fantaye FT Sene KM Damtew SA. Determinants of fertility desire among reproductive aged married and cohabiting women in Ethiopia a multilevel analysis of PMAET 2021 data. Sci Rep. (2025) 15(1):1–12. 10.1038/s41598-025-22976-2 41214103 PMC12603104

[24] Tessema ZT Yazachew L Tesema GA Teshale AB. Determinants of postnatal care utilization in sub-Saharan Africa: a meta and multilevel analysis of data from 36 sub-Saharan countries. Ital J Pediatr. (2020) 46:1–11. 10.1186/s13052-020-00944-y 33246475 PMC7693498

[25] Cameron AC Trivedi PK. Regression Analysis of Count Data. New York: Cambridge University Press (2013).

[26] Getaneh FB Belete AG Ayres A Ayalew T Muche A Derseh L. OPEN A generalized Poisson regression analysis of determinants of early neonatal mortality in Ethiopia using 2019 Ethiopian mini demographic health survey. Sci Rep. (2024) 14:1–9. 10.1038/s41598-024-53332-5 38307953 PMC10837113

[27] Billari FC Giuntella O Stella L. Discussion paper series does broadband internet affect fertility? Does broadband internet affect fertility? (2017) (10935).

[28] Sani J Halane S Ahmed AM Ahmed MM. Application of machine learning algorithms and SHAP explanations to predict fertility preference among reproductive women in Somalia. Sci Rep. (2025) 15(1):1–16. 10.1038/s41598-025-04704-y 40685342 PMC12277441

[29] Abdikarim H Abdirashid H Hussein MA Yousuf AM Muse AH Nadarajah S. A multinomial logistic regression analysis of fertility decisions and associated determinants among reproductive age women in Somaliland: utilization of 2020 SLHDS data. Women's Heal Reports. (2025) 6(1):731–41. 10.1177/26884844251365809 PMC12413255 40917697

[30] Adam WM Said Osman AM Jama YM Mohamud MA Omar SI Abshirow HA. Factors associated with infertility among women attending reproductive health centers in Mogadishu, Somalia: a cross-sectional study. Int J Gen Med. (2026) 7074:1–10. 10.2147/IJGM.S516562 PMC13007688 41878617

[31] Tessema ZT Azanaw MM Bukayaw YA Gelaye KA. Geographical variation in determinants of high-risk fertility behavior among reproductive age women in Ethiopia using the 2016 demographic and health survey: a geographically weighted regression analysis. Arch Public Heal. (2020) 78(1):1–12. 10.1186/s13690-020-00456-5 PMC7436963 32832078

[32] Ahmed MM Omar MA Sheikh Y Hassan A Ali AS. Determinants of children ever born among women in Somalia: a national survey study. Arch Public Health. (2026) 84:1–19. 10.1186/s13690-026-01980-6 PMC13450494 42231495

[33] Silverman BW. Density estimation for statistics and data analysis. (1986) (1951):1–22. 10.1201/9781315140919

[34] Hilbe JM. Negative Binomial Regression. Cambridge: Cambridge University Press (2011).

[35] Aike HAI. A new look at the statistical model identification (1974) AC-19:716–23.

[36] Schwarz G. Estimating the dimension of a model. Ann Statist. (1978) 6(2):461–4. 10.1214/aos/1176344136

[37] StataCorp LLC. Stata: Release 17. Statistical Software. College Station: Stata Press (2021).

